# 

**Published:** 1900-05

**Authors:** 


MARRIAGE.

On April 26, 1900, at Mt. Joy, Lancaster County, Pa., Dr. E. W. Newcomer, graduate of class of '99, Veterinary Department of the University of Pennsylvania, was married to Miss Minnie Brenneman. The happy couple made a tour of Baltimore, Washington, Richmond, and returned by way of Philadelphia.

Secretary.

### PERSONALS.

Dr. H. A. Meisner, of Baltimore, Md., the dark horse in the race for State Veterinarianship of Maryland, wears a sanguine smile as he basks in the confidence of carrying this trophy in his belt.

Dr. Ernest Spaeth, of Wyoming, who was recently on a visit to his home in Philadelphia, has returned again to the region of the setting sun.

Editor Hoskins, of the Journal, will be one of the Democratic electors of Pennsylvania, and only wishes that his State was not so overwhelmingly Republican that he could anticipate the casting of a vote in the Electoral College for W. Jennings Bryan.

Dr. Duncan McEachran, of Montreal, has purchased over five hundred range-horses in Canada for the English army in the South African war.

Dr. H. L. Ramacciotti has been appointed City Veterinarian and food-inspector for Omaha, Neb., for a term of three years. A well-deserved appointment.

The famed "Pimlico" races of "Maryland, My Maryland," continue to woo the attendance of our esteemed colleague, Dr. William Dougherty, of Baltimore.

Dr. M. Jacobs, resident surgeon of the Veterinary Department of the University of Pennsylvania, was among those who were before the examining board for inspector in the Bureau of Animal Industry.

Professor Joseph Clarke Robert, B.S., V.M.D., A. and M. College, Starkville, Mississippi, received the degree of M.D. from Sewanee Medical College, University of the South, January 18, 1900.

Dr. Charles H. Higgins has been transferred from the cattle quarantine system of the Dominion of Canada to that of the public health, and is engaged in establishing a bacteriological and biological laboratory at Victoria, B. C., where biologic products will be produced for the benefit of bubonic plague appearing on that coast.

Dr. W. W. Martin, of Battery A, and for a time in the army service in Cuba, but more recently engaged in commercial enterprises, was a visitor to Philadelphia in April, and spent several days at the veterinary department, where for a time he filled the rôle of resident surgeon.

Dr. D. S. White, of Columbus, Ohio, was presented with a handsome set of harness by his class of horseshoers receiving instructions under his teaching the past winter.

Dr. Charles F. Dawson, late of the Bureau of Animal Industry, has accepted a position in the laboratory of Parke, Davis & Co., Detroit, Mich.

Dr. H. S. Drake, of Leesburg, Va., has been on the sick list since the beginning of the New Year.

Dr. T. B. Pote, formerly of Terre Haute, Ind., is now located at St. Louis, Mo.

Much credit is given Professor A. Smith, of Toronto, for the successful outcome of the Toronto horseshow.

Dr. C. C. Cattanach, of New York, is keenly interested in the saddle horse contest now being conducted by the *Rider and Driver*, and names " Ladysmith " as his first choice.

Dr. A. McKenzie, of Washington, D. C., has entered the field of proprietary remedies, and now uses the columns of sporting papers in advertising the same.

Dr. Thomas W. Scott, of Clarksville, Tenn., entered the service of the Bureau of Animal Industry in April and was assigned to duty in Kansas City, Mo.

Dr. N. L. Townsend, of Moorestown, N. J., was one of the applicants before the civil-service examining board for meat-inspector in April.

Dr. V. G. Hunt, of Arcola, Ill., has turned the sixty-sixth milestone in life, is still active in professional life, keenly interested in Association work, and continues to read three of the leading veterinary journals in the search for fresh knowledge and new truths.

Dr. H. C. Glover, of New York City, has been elected president of the Patchogue Long Island Driving Association.

Professor H. C. Wood, of Philadelphia, well known to every veterinary student of the University of Pennsylvania, has been chosen as one of the authorities to revise the United States *Pharmacopœia.*

Veterinarian Major Phillips, of the British Army, has been sojourning in Canada for a period, engaged in the purchase of remounts for the British cavalry service.

Dr. James L. Robertson, of New York City, will be one of the veterinarians of the Elk Ridge horseshow, Baltimore, in May.

Dr. H. G. Patterson, of St. Joseph, Mo., Hal C. Simpson, of Denison, Iowa, and D. B. Pierce, of Springfield, Mass., have accepted service with the British Government and shipped from New Orleans to South Africa with consignments of horses.

Drs. Salmon, Huidekoper, Hoskins, and Clement, of the Army Legislative Committee, and President Pearson, of the A. V. M. A., were in conference at Washington, D. C., in April relative to the progress of army legislation.

Dr. S. Beattie, of Madison, Wis., was on the sick list in April.

Dr. H. James Elliott, of Brandon, Manitoba, is the secretary of the Brandon Kennel Club; takes a very active interest in the field trials of the canine species and event-shows of their organization. The club is in a flourishing condition, and contributes very much to the increased interest in the canine world through Manitoba.

Dr. S. G. Burkholder, of Chicago, Ill., severed his connection with the Bureau of Animal Industry in order to attend the Northwestern Medical College, from which institution he expects to graduate this spring.

Dr. C. S. Gelbert, of Scranton, Pa., was a visitor to the Journal office and Veterinary Department of the University of Pennsylvania in May.

City Veterinarian Ramacciotti, of Omaha, will be at Detroit, there to discuss food-inspection problems and further this important work over our land.

